# Supplementary material for: Structural basis for +1 ribosomal frameshifting during EF-G-catalyzed translocation
Source: Nat Commun. 2021 Jul 30;12:4644. doi: 10.1038/s41467-021-24911-1 (PMC8324841; doi:10.1038/s41467-021-24911-1)
Supplement: Supplementary file 1 — Supplementary information [file 41467_2021_24911_MOESM1_ESM.pdf]

SUPPLEMENTARY INFORMATION

Structural basis for +1 ribosomal frameshifting during EF-G-catalyzed translocation

Gabriel Demo[1,2], Howard B. Gamper[3], Anna B. Loveland[1], Isao Masuda[3], Egor Svidritskiy[1], Ya-Ming Hou[3,*], Andrei A. Korostelev[1,*]

[1]RNA Therapeutics Institute, Department of Biochemistry and Molecular Pharmacology, UMass Medical School, Worcester, MA, USA

[2]Central European Institute of Technology, Masaryk University, Kamenice 5, Brno, 625 00, Czech Republic

[3]Department of Biochemistry and Molecular Biology, Thomas Jefferson University, Philadelphia, PA, USA

[*]Correspondence: Ya-Ming.Hou@jefferson.edu or Andrei.Korostelev@umassmed.edu

Contains:

Supplementary Tables 1-2

Supplementary Figures 1-9

Supplementary Table 1. Primers used for mutagenesis to insert a slippery or non-slippery motif at the second position of the lacZ plasmid.

Description	Sequence (5′ to 3′)
Forward for "CCC-A" variant	ATGCCCAACCATCATTACGCCAAG
Forward for "CCA" variant	ATGCCAACCATCATTACGCCAAG
Forward for "CCA-A" variant	ATGCCAAACCATCATTACGCCAAG
Reverse for all variants	GAATTCTGTTTCCTGTGTGAAATTGTTATCC

Supplementary Table 2. Refinement statistics for cryo-EM structures of non-frameshifting and frameshifting complexes.

	I	II	III	I-FS	I^{rot}-FS	II-FS	III-FS
PDB	7K50	7K51	7K52	7K53	7LV0	7K54	7K55
EMDB	EMD-22669	EMD-22670	EMD-22671	EMD-22672	EMD-23528	EMD-22673	EMD-22674
**Data collection and processing**							
Magnification	130000x	130000x	130000x	130000x	105000x	130000x	130000x
Voltage (kV)	300	300	300	300	300	300	300
Electron exposure ($e^-/Å^2$)	47.5	47.5	47.5	47.5	40.2	47.5	47.5
Defocus range (µm)	0.8-2.0	0.8-2.0	0.8-2.0	0.8-2.0	0.8-2.0	0.8-2.0	0.8-2.0
Super-resolution pixel size (Å)	0.525	0.525	0.525	0.525	0.415	0.525	0.525
Symmetry imposed	C1	C1	C1	C1	C1	C1	C1
Initial particle images (no.)	62,716	62,716	62,716	164,504	178,117	164,504	164,504
Final particle images (no.)	4,263	3,179	4,612	12,108	3,658	9,059	6,029
Map resolution (Å)***	3.4	3.5	3.4	3.2	3.2	3.2	3.3
FSC threshold	0.143	0.143	0.143	0.143	0.143	0.143	0.143
**Refinement**							
Initial model used (PDB code)	5UYM	5UYM	5UYM	5UYM	5UYM	5UYM	5UYM
Model resolution (Å)	3.2	3.2	3.2	3.2	3.2	3.2	3.2
Correlation Coefficient (cc_mask)*	0.829	0.791	0.807	0.842	0.804	0.815	0.800
Real space R-factor †	0.224	0.250	0.242	0.224	0.234	0.244	0.256
Map sharpening *B* factor ($Å^2$)	-80	-80	-80	-80	-80	-80	-80
Model composition*							
Non-hydrogen atoms	149700	153371	153371	149698	147330	153369	153369
Protein residues	5944	6629	6629	5944	5864	6629	6629
RNA residues	4811	4732	4732	4811	4734	4732	4732
Mg^{2+}/GDPCP	0/0	1/1	1/1	0/0	0/0	1/1	1/1
B factors ($Å^2$)*							
Protein	146.4	165.9	169.4	139.6	112.11	146.1	152.0
RNA	146.4	152.0	161.1	135.1	108.92	134.6	140.6
R.m.s. deviations*,§							
Bond lengths (Å)	0.010	0.012	0.011	0.009	0.012	0.011	0.012
Bond angles (°)	1.125	1.252	1.224	1.100	1.250	1.167	1.258
Validation**							
MolProbity score	2.64	2.84	2.73	2.55	2.94	2.74	2.85
Clashscore	19.22	25.11	21.37	17.92	28.79	22.40	24.93
Poor rotamers (%)	1.87	2.08	1.88	1.52	3.85	1.81	2.17
Ramachandran plot**							
Favored (%)	85.09	81.68	82.71	84.77	80.30	84.33	82.60
Allowed (%)	13.21	16.48	15.89	14.08	17.75	14.06	15.77
Disallowed (%)	1.70	1.84	1.40	1.15	1.95	1.61	1.63
Validation (RNA)**							
Good sugar pucker (%)	99.4	99.5	99.5	99.4	99.5	99.5	99.4
Good backbone (%)	87.5	85.5	88.1	87.9	84.6	86.8	86.9

*** from Frealign (FSC_part)
** from Molprobity
* from Phenix
† from RSRef
§ root mean square deviations

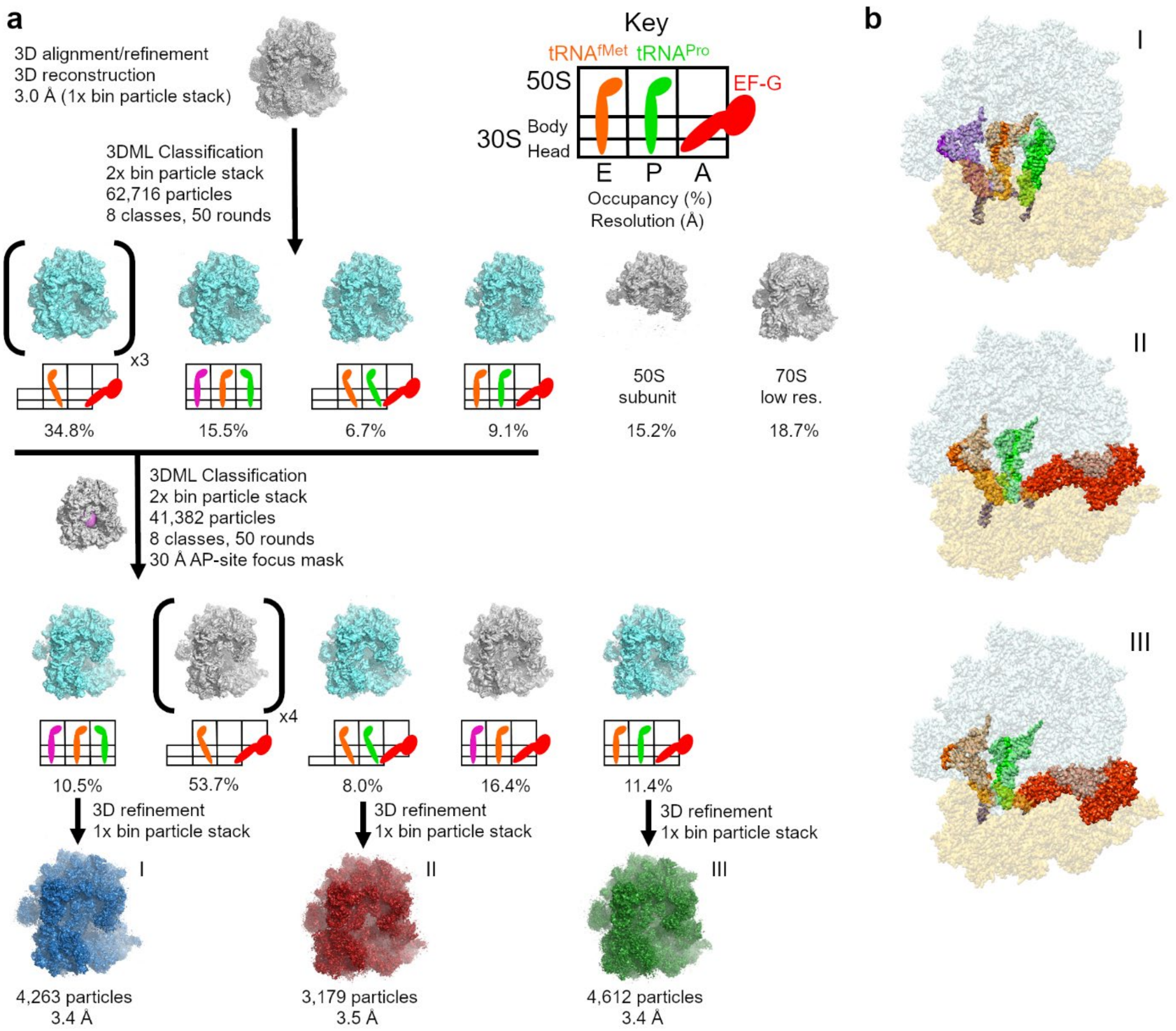

Supplementary Fig. 1. Scheme of maximum-likelihood classification resulting in cryo-EM maps of 70S ribosomes bound with and without EF-G for the non-frameshifting complex. (**a**) Classification of the dataset obtained for 70S ribosomes with the non-frameshifting CCA-A mRNA. For each sub-classification the used spherical mask is highlighted (pink sphere). (**b**) Segmented cryo-EM maps corresponding to Structures I, II, and III. The maps are colored as in Fig. 3.

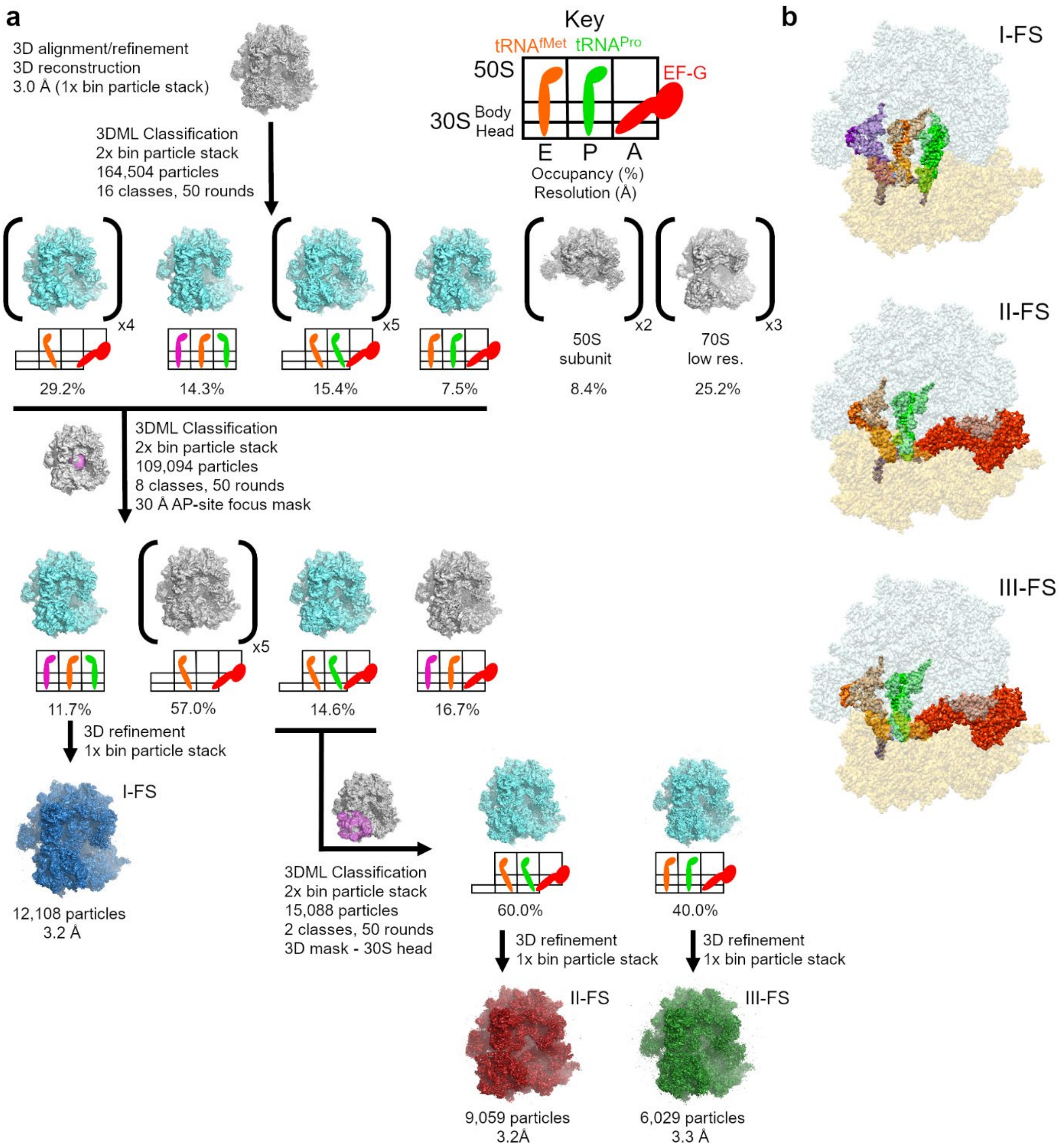

Supplementary Fig. 2. Scheme of maximum-likelihood classification resulting in cryo-EM maps of 70S ribosomes bound with and without EF-G for the frameshifting complex. (**a**) Classification of the dataset obtained for 70S ribosomes with the frameshifting CCC-A mRNA. For each sub-classification the used spherical or 3D mask is highlighted (pink sphere or region). (**b**) Segmented cryo-EM maps corresponding to Structures I-FS, II-FS, and III-FS. The maps are colored as in Fig. 3.

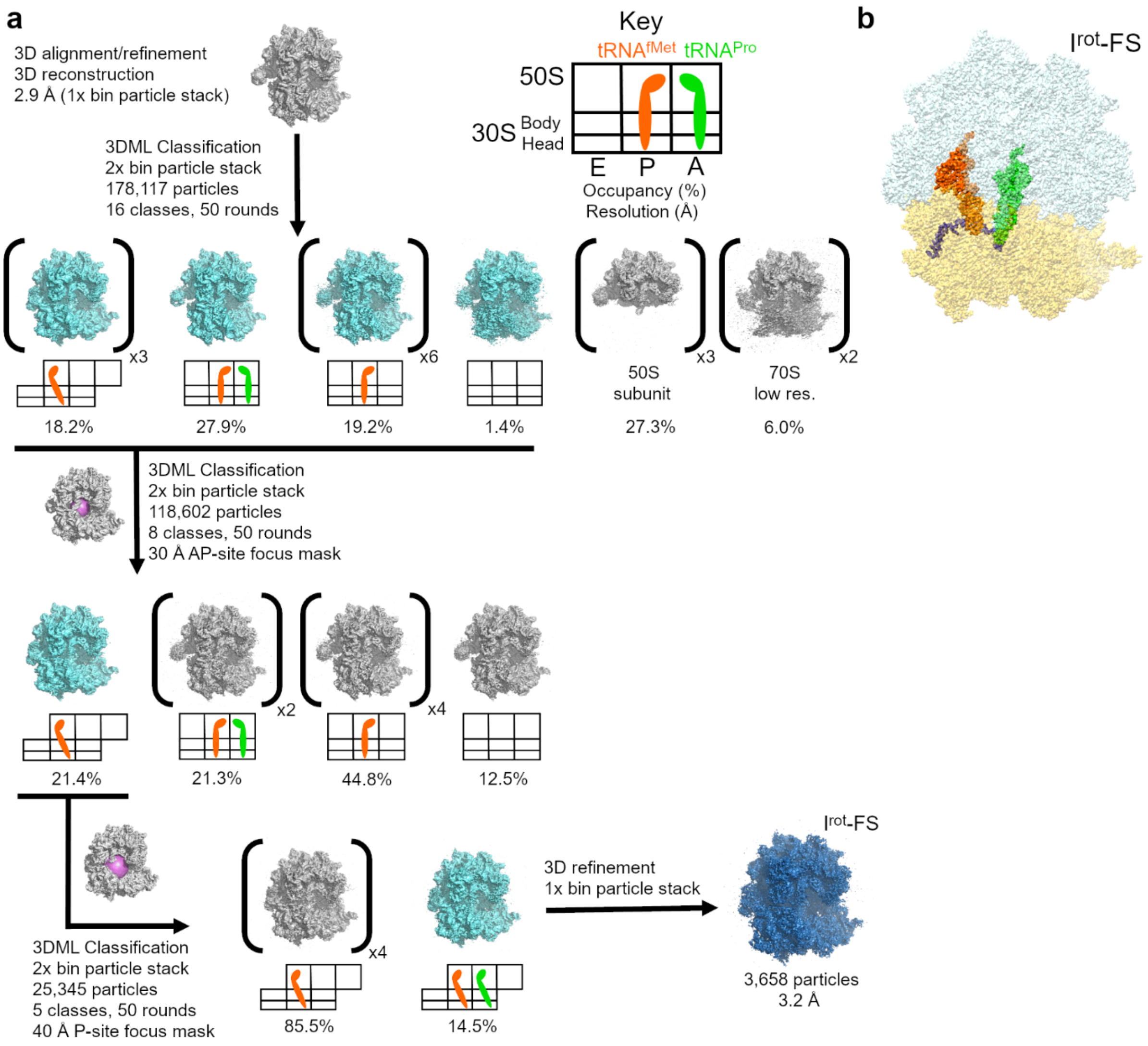

Supplementary Fig. 3. Scheme of maximum-likelihood classification resulting in cryo-EM map of 70S rotated pre-translocation ribosomes bound with 2 tRNAs for the frameshifting complex. (**a**) Classification of the dataset obtained for 70S ribosomes with the frameshifting CCC-A mRNA. For each sub-classification the used spherical mask is highlighted (pink sphere). (**b**) Segmented cryo-EM map corresponding to Structure I^{rot}-FS. The map is colored as in Fig. 3.

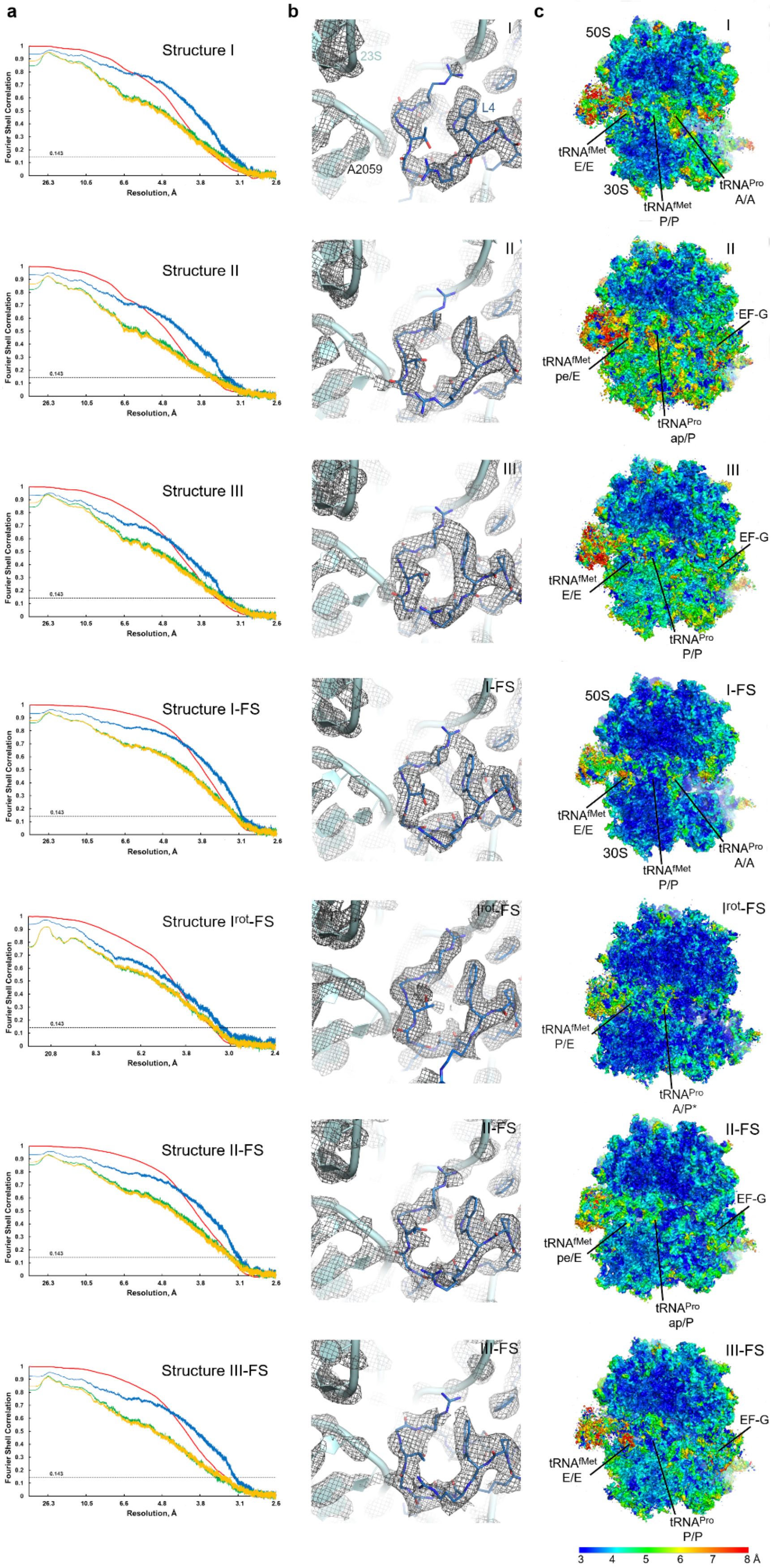
a
b
c
Structure I
Structure II
Structure III
Structure I-FS
Structure I^rot-FS
Structure II-FS
Structure III-FS
Fourier Shell Correlation
Resolution, Å
0.143
23S
L4
A2059
I
II
III
I-FS
I^rot-FS
II-FS
III-FS
50S
30S
tRNA^fMet E/E
tRNA^fMet P/P
tRNA^Pro A/A
EF-G
tRNA^fMet pe/E
tRNA^Pro ap/P
tRNA^Pro P/P
tRNA^fMet P/E
tRNA^Pro A/P*
3
4
5
6
7
8 Å

Supplementary Fig. 4. Global and local resolution for non-frameshifting and +1 frameshifting complexes. (**a**) Fourier shell correlation (FSC) between even- and odd-particle half maps (red) show that map resolutions range from 3.4 to 3.5 Å for Structures I-III and 3.2 to 3.3 Å for Structures I-FS-III-FS (at FSC = 0.143, dotted line); FSC between final models and final maps (blue), and cross-validation half-maps (half-map 1 in green and half-map 2 in yellow) masked FSCs are also shown. (**b**) Examples of local map resolution for rRNA and protein in the vicinity of the peptidyl transferase center are shown for each structure (23S rRNA and protein L4 are labeled for reference). (**c**) Local resolutions in cryo-EM densities for Structures I-III and Structures I-FS-III-FS, calculated using Blocres. Slab views show the ribosome interior in the vicinity of tRNAs and EF-G.

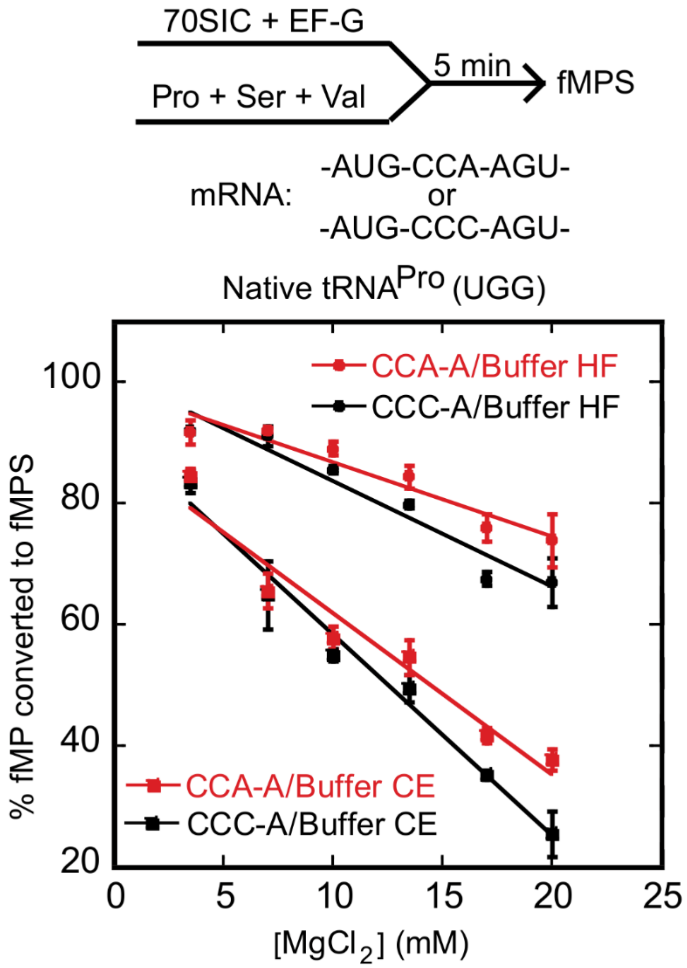

Supplementary Fig. 5. Fractional conversion of fMP to fMPS with native *E. coli* tRNA^{Pro}(UGG) on the non-slippery CCA-A mRNA (red) or the slippery CCC-A (black) mRNA, measured in the HF or CE buffers at 20 °C is reported as a function of $MgCl_2$ concentration. The bars in the graphs are SD of three (n = 3) independent experiments, and the data are presented as mean values ± SD.

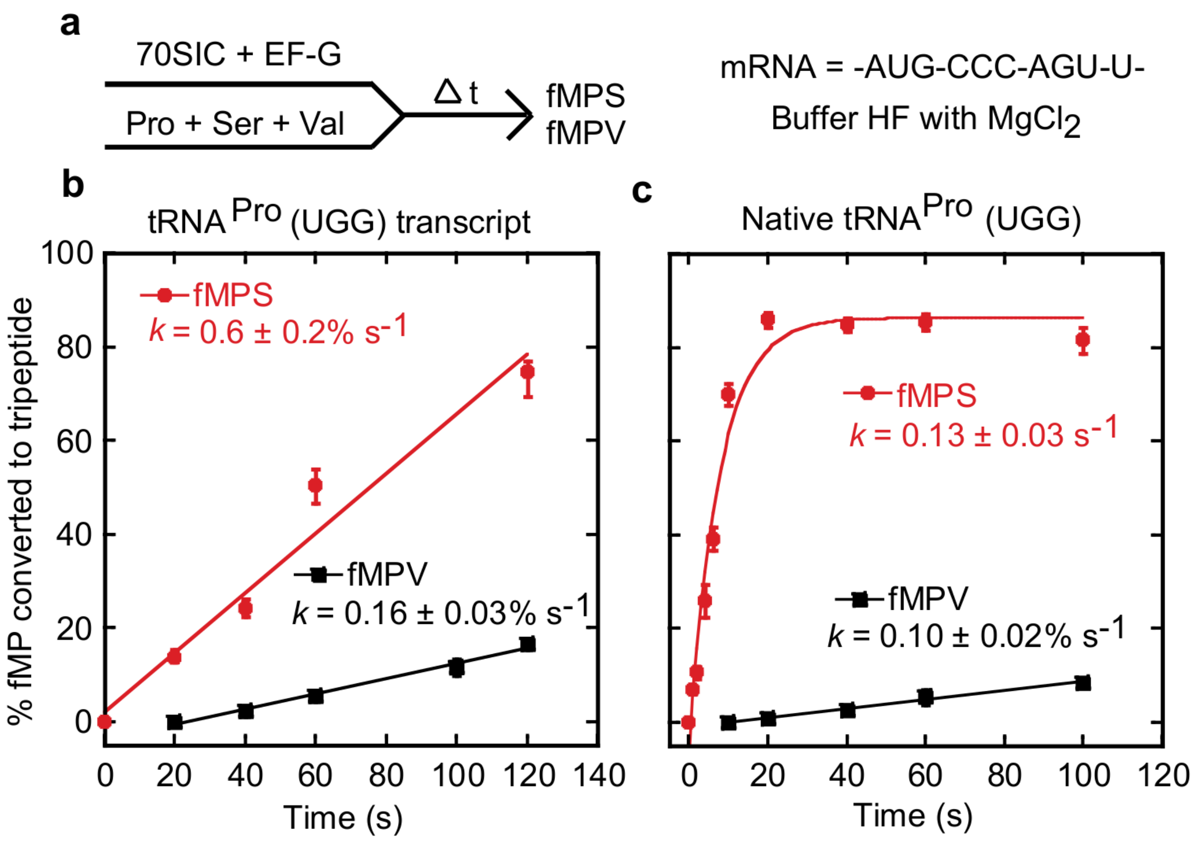

Supplementary Fig. 6. Time progress of fractional conversion of fMP to fMPS or to fMPV with *E. coli* tRNA^{Pro}(UGG) on the slippery CCC-A mRNA. (**a**) An *E. coli* 70SIC was rapidly mixed with an equal molar mixture of TCs of *E. coli* tRNA^{Pro}(UGG), Ser-tRNA^{Ser}, and Val-tRNA^{Val} to measure the fraction conversion in the HF buffer with 3.5 mM $MgCl_2$ at 20 °C. (**b**) The fractional conversion of fMP to fMPS (red) or to fMPV (black) for the transcript-state of *E. coli* tRNA^{Pro}(UGG), showing a slow and incomplete conversion of fMP to fMPS, and the rate of conversion to fMPV. (**c**) The fractional conversion of fMP to fMPS (red) or to fMPV (black) for the native-state of *E. coli* tRNA^{Pro}(UGG), showing a rapid and stoichiometric conversion of fMP to fMPS, and the rate of conversion to fMPV. The bars in the graphs are SD of three (n = 3) independent experiments, and the data are presented as mean values ± SD.

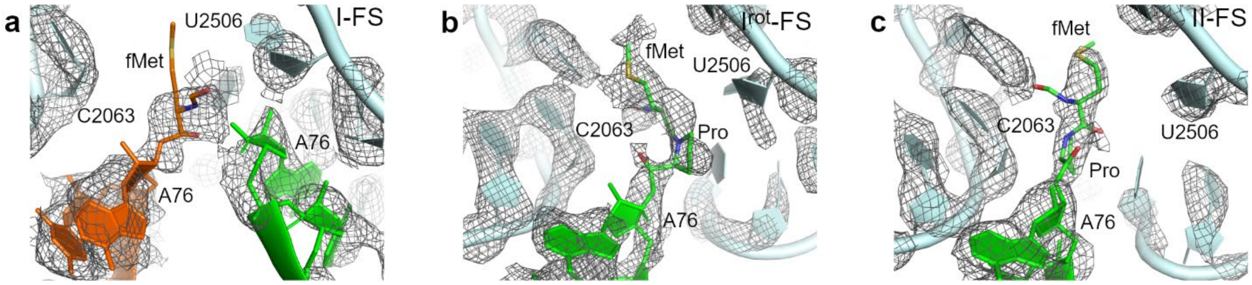

Supplementary Fig. 7. Cryo-EM density (mesh) of the peptidyl transferase center of the frame-shifting structures in the pre-translocation (**a**, I-FS), (**b**, I^{rot}-FS) and EF-G-bound translocation (**c**, II-FS) states. In the pre-translocation state (in both Structure I and I-FS, shown in panel **a**, density does not allow unambiguous interpretation of the peptidyl-transfer states of the amino acids fMet and Pro, suggesting a mixture of aminoacyl- and dipeptidyl-tRNA states. fMet was modeled in both structures, because continuous density is observed between the P-tRNA nucleotide A76 and the amino-acyl moiety. The maps (gray mesh) were sharpened by applying the B-factor of -80 Å^2 and are shown at 2.5 σ. The 50S ribosomal subunit and tRNAs are colored as in Fig. 3.

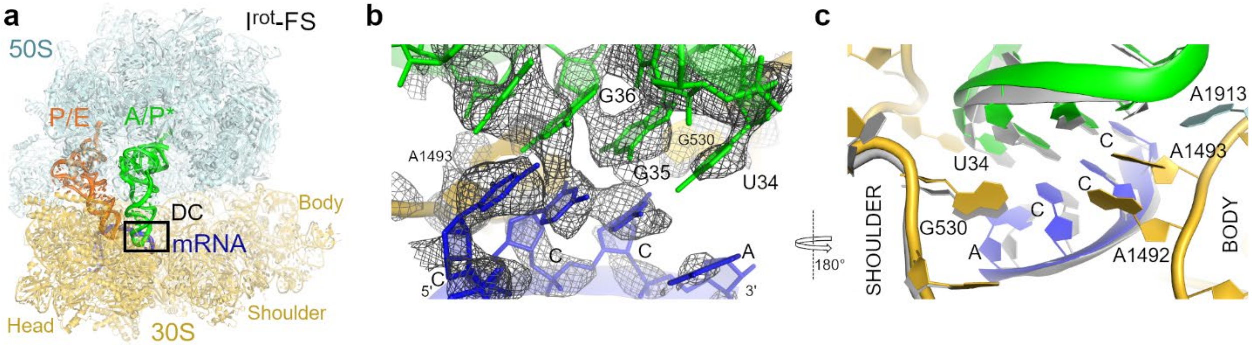

Supplementary Fig. 8. Cryo-EM structure of a rotated pre-translocation 70S ribosome with the frameshifting mRNA and Pro-tRNA^{Pro} in the A/P* and fMet-tRNA^{fMet} in the P/E states. (**a**) Overall view of the 70S structure with frameshifting mRNA (CCC-A; Structure I^{rot}-FS). (**b**) Cryo-EM density (gray mesh) for codon-anticodon interaction between frameshifting mRNA and tRNA^{Pro} in the A site of Structure I^{rot}-FS. The view approximately corresponds to the boxed decoding center region (DC) in panel **a**. The map was sharpened with a B-factor of -80 Å^2 and is shown at 2.5 σ. (**c**) Decoding center nucleotides G530 (in the shoulder region) and A1492-A1493 (in the body region) stabilize the codon-anticodon helix in Structure I^{rot}-FS. Conformation of the decoding center in Structure I^{rot}-FS is similar to that in Structure I-FS (16S, mRNA and Pro-tRNA^{Pro} shown in gray). Structural alignment was obtained by superposition of the body of 16S ribosomal RNAs. The ribosomal subunits, tRNAs and mRNA are colored as in Fig. 3.

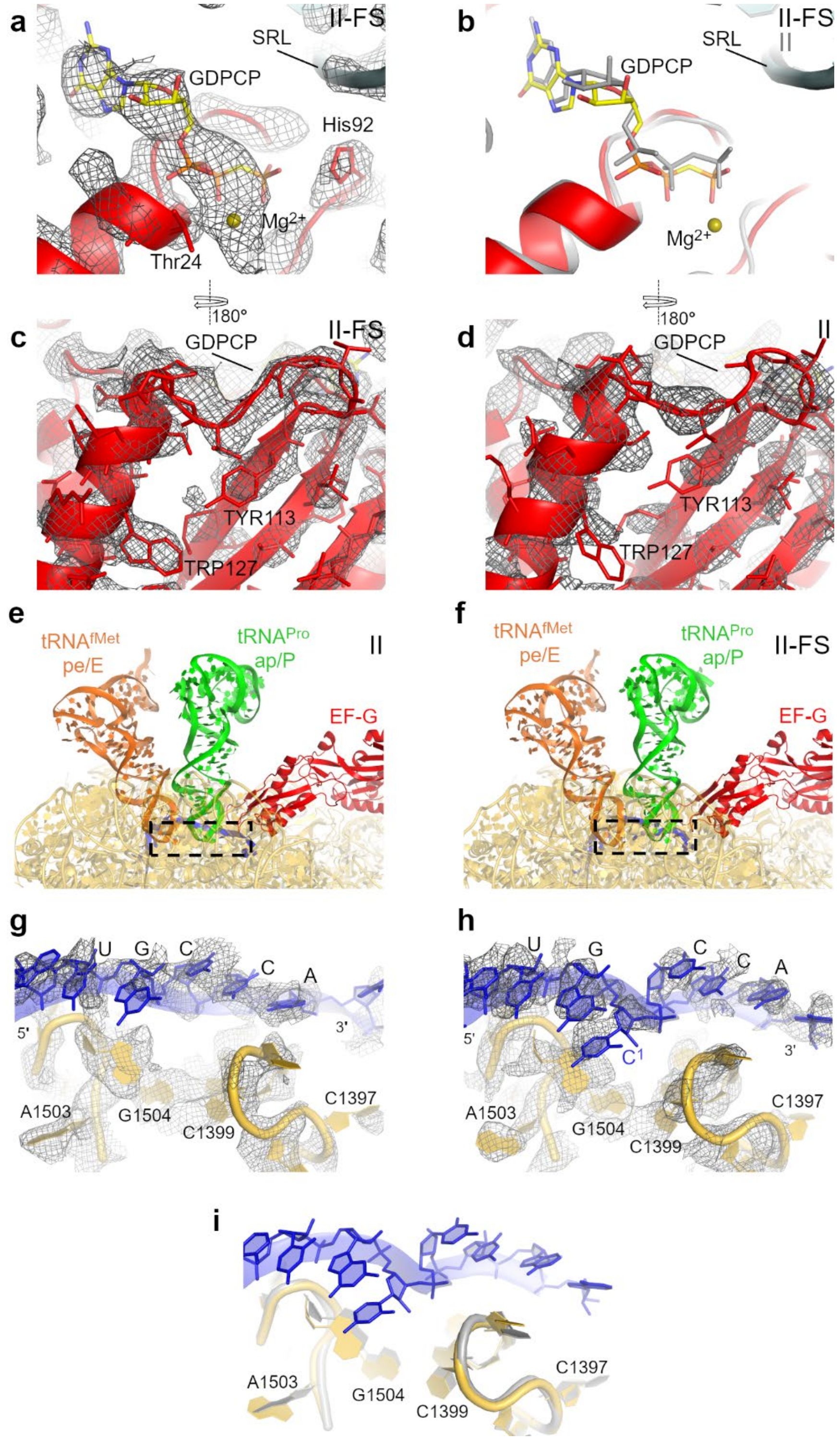

Supplementary Fig. 9. **The GTPase region of EF-G and 16S rRNA surrounding the E and A sites in the EF-G-bound translocation complexes.** (**a**) Cryo-EM density (gray mesh) of the EF-G GTPase center with GDPCP in structure II-FS. The map is sharpened by applying the B-factor

of -80 Å^2 and is shown at 2.5 σ. (**b**) Structural alignment of the GTPase center (red, II-FS) and GDPCP (yellow, II-FS) with those of the non-frameshifted structure (gray, II). Structural alignment was performed by superposition of 23S rRNAs. (**c**-**d**) Cryo-EM densities (gray mesh, at 2.5 σ) for the GTPase domain I of EF-G in Structures II-FS and II. (**e**-**i**) Comparison of mRNA-binding regions of the 16S rRNA in Structures II and II-FS; cryo-EM densities (gray mesh, at 2.5 σ) are shown in panels **g** and **h**, alignment of structures II-FS and II (gray) is shown in panel **i**. This comparison shows similar positions of the region containing nucleotides C1397 and A1503 proposed to participate in mRNA frame maintenance (see Results). Structural alignment was performed by superposition of 16S rRNAs. The ribosomal subunits, tRNAs, mRNA and EF-G are colored as in Fig. 4.
